# Metachronous pulmonary and adrenal metastases after liver transplantation for hepatocarcinoma

**DOI:** 10.1186/1477-7819-9-156

**Published:** 2011-11-28

**Authors:** Shan-Wen Chen, Shuo Wang, Bo Wang, Wei-Dong Li, Sheng Yan, Li-Ping Xie

**Keywords:** liver transplantation, metachronous, metastasis, surgical management

## Abstract

**Background:**

The worldwide experience of surgical resection for isolated metastasis following liver transplantation (LT) for hepatocellular carcinoma (HCC) is limited.

**Methods:**

The case of a 60-year-old patient performed successful surgical management for metachronous pulmonary and adrenal metastases from HCC after LT.

**Results:**

Eighty months after LT, he was presently alive and disease-free with a normal AFP value.

**Conclusion:**

The case is an interesting report on a somehow indolent metastatic spread of HCC after LT. It should be considered that metachronous metastatic resectable disease, with no data of recurrence at the primary site in an operable patient, is an indication to perform a surgical resection.

## Background

The worldwide experience of surgical resection for isolated metastasis following liver transplantation (LT) for hepatocellular carcinoma (HCC) is limited [1-3]. Here we reported a rare case of successful surgical management of metachronous pulmonary and adrenal metastases after LT for HCC. To our knowledge, successful managements for metachronous pulmonary and adrenal metastases from HCC after LT have not been previously reported in English literature.

## Case presentation

A 60-year-old man, with a 20-year history of type B hepatitis and hepatic cirrhosis, presented with a solid large mass in the left lateral segment. The patient was poor-hepatic functional reserve because of atrophy of the right liver and compensative hyperplasia of the left liver according to imaging studies. The serum alpha-fetoprotein (AFP) value was 7,812.0 ng/ml, serum albumin level was 29.4 g/L, and other laboratory data were within normal limits. The Child-Pugh classification of the cirrhosis was Child early B cirrhosis. He underwent LT on 9 February 2004 and had received 3 cycles' postoperative adjuvant chemotherapy with capecitabine and oxaliplatin for 5 months. After LT, the AFP value decreased within a normal range and stayed normal for thirty months.

Thirty-three months after LT, a 3.0 cm×3.5 cm lesion was detected in the right lung with elevated AFP value (167.1 ng/ml). Because positron emission tomography (PET) scan revealed no fluorodeoxyglucose (FDG)-avid focus in transplanted liver and other extrahepatic organs, partial resection of the lower lobe of the right lung was performed. After pulmonary resection, the AFP value decreased within a normal range.

**Figure 1:**
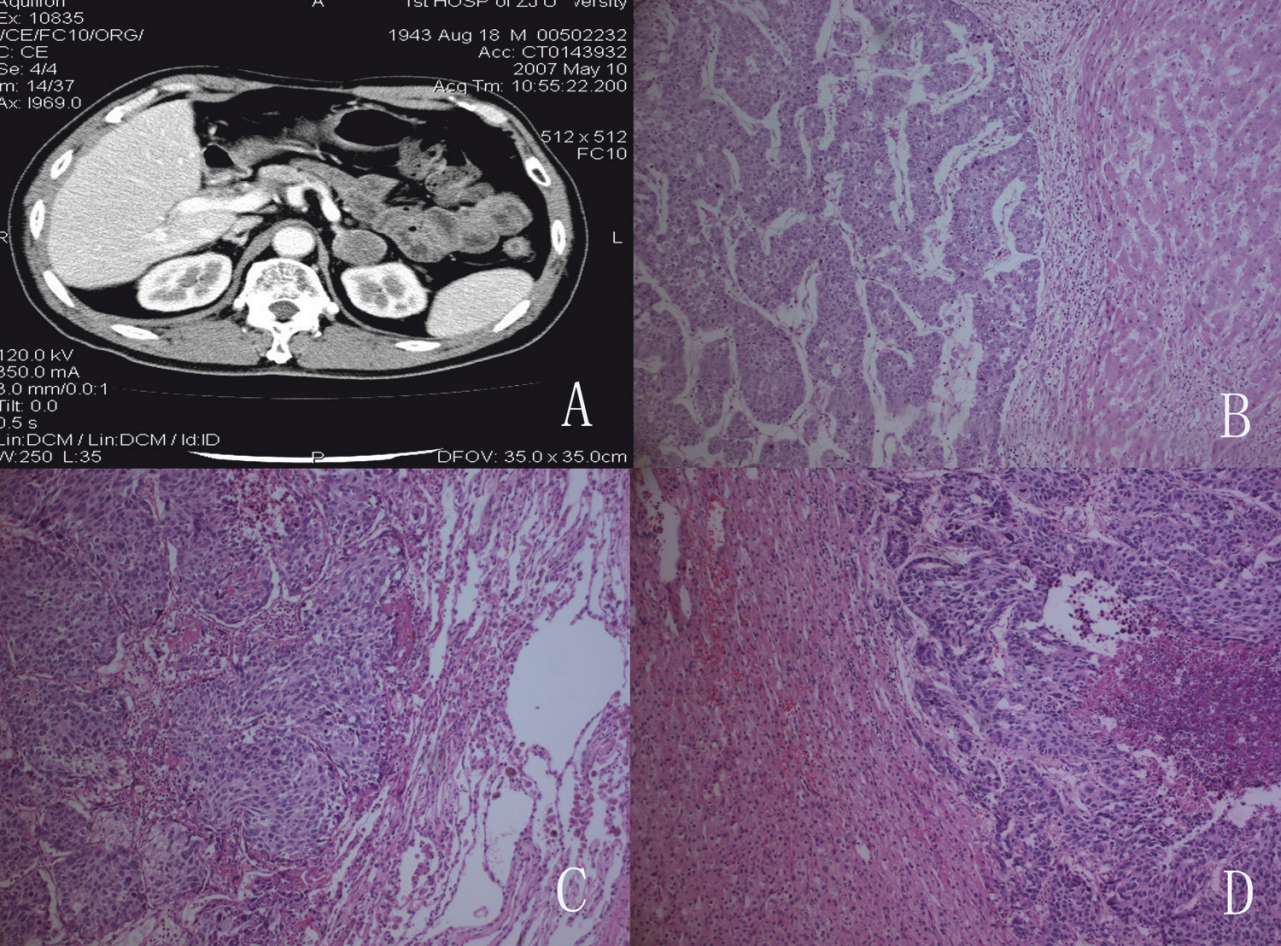
**Enhanced abdominal computed tomography**. *A*, Enhanced abdominal computed tomography revealed a homogeneous 4.0 cm×3.5 cm mass in the left adrenal gland with no calcification. *B*, The histological findings of the hepatic tumor. *C*, The histological findings of the lung tumor.*D*, The histological findings of the adrenal tumor (H&E, Stain×400).

**Figure 2:**
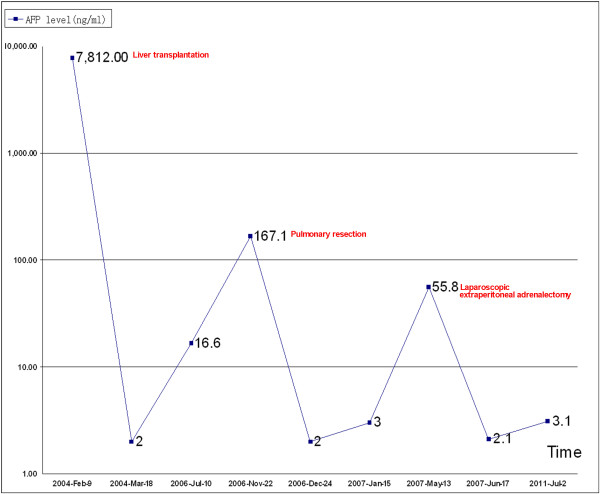
**The patient's AFP level**. The patient's AFP level was closely correlated to tumor metastases and therapeutic interventions.

Thirty-nine months after LT, an abdominal computed tomography (CT) scan revealed a 4.0 cm×3.5 cm homogeneous mass in the left adrenal gland with elevated AFP value (55.8 ng/ml) (Figure 1A). Furthermore, the PET scan revealed a FDG-avid enlarged left adrenal gland without FDG-avid activity in transplanted liver. Neither recurrent nor metastatic foci in any other organs was detectable after thorough examination, except for the left adrenal grand. Review of the older PET scan and other imaging studies showed no evidence of the adrenal lesion. The American Society of Anesthesiologists (ASA) grade of the patient was II grade. Based on the previous findings, a laparoscopic extraperitoneal adrenalectomy was performed on 13 May 2007. Postoperatively, the patient received 6 cycles' adjuvant chemotherapy with gemcitabine and 5-fuorouracil. Three years after adrenalectomy, the patient is presently alive and disease-free with a normal AFP value. He is presently on tacrolimus and sirolimus for immunosuppression. The tumor metastases and therapeutic interventions related with the changes of the AFP levels could be seen in Figure 2.

## Histopathology

The histopathology of the resected liver revealed a large tumor in the left lobe (9 cm×8 cm×8 cm) with features of poorly differentiated hepatocellular carcinoma. The uninvolved liver showed cirrhosis (Figure 1B). The tumors in the lung and adrenal were 3.0 cm×3.0 cm× 3.5 cm and 3.0 cm×3.3 cm× 4.0 cm in size, respectively. The histological findings of the lung tumor showed that cells with round nuclei proliferated in clusters, and mitoses were visible in many nuclei (Figure 1C). In the adrenal gland, well-defined oval cells or nuclei proliferated solidly, and necrotic tissue was visible in the cancerous lesion (Figure 1D). AFP-positive cells were identified by immunohistochemistry both in the lung and adrenal lesions.

## Discussion

LT is claimed to cure HCC and the underlying cirrhosis simultaneously in selected patients. Nevertheless, a careful follow-up is needed in those cases due to the possibility of neoplastic recurrence, which could take place not only in the graft, but also in extrahepatic organs such as lung, adrenal glands, and bone [4]. The cumulative survival rates of 6, 12, 24, and 36 months after the initial diagnosis of extrahepatic metastases were 44.1%, 21.7%, 14.2%, 7.1%, respectively. The median survival time was 4.9 months (range, 0-37 months) [5]. Extrahepatic metastasis is a common cause of deaths in patients after LT. Those patients, who usually have multiple metastases, could only be offered supportive care or palliative chemotherapy. However, previous studies showed that a solitary metastasis might have the opportunity of surgical resection [1,6,7]. In a report from Lyon, there were 7 patients who underwent adrenalectomy for metastatic HCC, two died in the postoperative period in relation with pulmonary embolism (n = 1) or acute pancreatitis (n = 1), and the mean survival time of the other five patients were 38 months after adrenalectomy [8].

The worldwide experience of surgical resection for isolated metastasis following LT for HCC is limited to case reports [1-3]. Rubio E et al reported one patient underwent adrenalectomy for the right adrenal metastasis 3 years after LT for HCC. The patient was alive when reported and disease-free for 24 months after adrenalectomy [1]. Herein, we have described a rare case of successful surgery for metachronous pulmonary and adrenal metastases after LT for HCC. Our patient had a unique character of metachronous metastasis instead of the systemic spread of cancer after operation. There were two metastatic sites: the lung and the adrenal gland; but each metastatic lesion was detectable only as a metachronous focus without any evidence of local recurrence or other extrahepatic metastasis. Furthermore, the patient's AFP level was closely corelated to tumor metastasis, which was a good indicator for tumor metastasis and made the surgical approach possible [9]. Palliative chemotherapy could be delivered to patients with recurrent HCC after LT with tolerable toxicity [10]. Capecitabine plus oxaliplatin regimen showed modest anti-tumour activity with tolerable toxicities in patients with advanced HCC [11,12]. Therefore, Capecitabine plus oxaliplatin regimen were followed routinely in patients with large and poorly differentiated hepatocellular carcinoma after LT in our centre. The patient is presently alive and disease-free for three years after the third operation. We hypothesize some potential beneficial elements, such as complete surgical removal, unusual sensitivity to adjuvant therapy [13], endocrine influence [14], allergic reaction [15], and interference with nutrition of the tumor [16]. We believe severe follow up is necessary for the patient in future.

## Conclusion

In conclusion, this is an interesting report on a somehow indolent metastatic spread of HCC. It should be considered that metachronous metastatic resectable disease, with no data of recurrence at the primary site in an operable patient, is an indication to perform a surgical resection.

## Consent

Written informed consent was obtained from the patient for publication of this case report and accompanying images. A copy of the written consent is available for review by the Editor-in-Chief of this journal.

## Abbreviations

LT: Liver transplantation; HCC: hepatocellular carcinoma; AFP: serum alpha-fetoprotein.

## Competing interests

The authors declare that they have no competing interests.

## Authors' contributions

SWC, SW, WDL, and SY participated in the admission and the care of this patient. All the authors participated in the conception, the design, data collection and interpretation, manuscript preparation and literature search. All authors have read and approved the final manuscript.
